# Comprehensive High-Resolution Analysis of the Role of an Arabidopsis Gene Family in RNA Editing

**DOI:** 10.1371/journal.pgen.1003584

**Published:** 2013-06-20

**Authors:** Stéphane Bentolila, Julyun Oh, Maureen R. Hanson, Robert Bukowski

**Affiliations:** 1 Department of Molecular Biology and Genetics, Biotechnology Building, Cornell University, Ithaca, New York, United States of America; 2 Computational Biology Service Unit, Cornell University, Ithaca, New York, United States of America; The University of Western Australia, Australia

## Abstract

In flowering plants, mitochondrial and chloroplast mRNAs are edited by C-to-U base modification. In plant organelles, RNA editing appears to be generally a correcting mechanism that restores the proper function of the encoded product. Members of the Arabidopsis RNA editing-Interacting Protein (RIP) family have been recently shown to be essential components of the plant editing machinery. We report the use of a strand- and transcript-specific RNA-seq method (STS-PCRseq) to explore the effect of mutation or silencing of every *RIP* gene on plant organelle editing. We confirm RIP1 to be a major editing factor that controls the editing extent of 75% of the mitochondrial sites and 20% of the plastid C targets of editing. The quantitative nature of RNA sequencing allows the precise determination of overlapping effects of RIP factors on RNA editing. Over 85% of the sites under the influence of RIP3 and RIP8, two moderately important mitochondrial factors, are also controlled by RIP1. Previously uncharacterized RIP family members were found to have only a slight effect on RNA editing. The preferential location of editing sites controlled by RIP7 on some transcripts suggests an RNA metabolism function for this factor other than editing. In addition to a complete characterization of the RIP factors for their effect on RNA editing, our study highlights the potential of RNA-seq for studying plant organelle editing. Unlike previous attempts to use RNA-seq to analyze RNA editing extent, our methodology focuses on sequencing of organelle cDNAs corresponding to known transcripts. As a result, the depth of coverage of each editing site reaches unprecedented values, assuring a reliable measurement of editing extent and the detection of numerous new sites. This strategy can be applied to the study of RNA editing in any organism.

## Author Summary

RNA editing is a co- or post-transcriptional RNA processing reaction that changes the nucleotide sequence of the RNA substrate. In flowering plants, mRNA editing is confined to organelle transcripts, altering cytidine to uridine. Recently, some members of a small Arabidopsis gene family were found to be important for editing of chloroplast and mitochondrial transcripts. Several methods have been developed to measure the amount of edited transcripts at specific Cs, but most of these methods either lack sensitivity or are unable to determine the number and location of edited Cs in a particular transcript. While sensitive assays have been previously developed, they are costly and labor-intensive precluding their use on a large-scale. In order to characterize the role of an entire gene family in RNA editing, we have successfully adapted RNA sequencing technology to characterize the effect of mutation and silencing of family members on organelle RNA editing. Our method to measure editing extent is sensitive, reliable, and cost-effective. As well as detecting additional family members that play a role in RNA editing, we have detected numerous new editing sites. Our strategy should benefit the investigation of RNA editing in any organism.

## Introduction

RNA editing alters the genetic information at specific sites on RNA molecules. Editing has been described in a wide range of organisms from viruses to animals and plants. Several systems involving unrelated mechanisms seem to have arisen separately during evolution [1]. In flowering plants, organelle mRNA transcripts are modified post-transcriptionally by C-to-U editing. 30 to 40 C-to-U editing events are typically found in flowering plant chloroplasts transcriptomes [2] and over 500 Cs are edited to U in Arabidopsis mitochondria [3]. Editing is an essential process that can correct deleterious mutations that would otherwise hamper the proper function of the encoded product, as some mutants impaired in editing die at the young seedling stage [4]. Site specificity of the C to be edited in plant organelle requires a *cis* sequence primarily upstream of the C and trans-factors that recognize the *cis*-element. Current information about *cis*-acting elements in plant organelles has been obtained by electroporation of mutated transcripts into isolated mitochondria, by analysis of editing of exogenous RNAs in either chloroplast or mitochondrial extracts, and by incorporation of altered genes into plastid genomes [5]–[8]. Experiments in both organelles have delineated the *cis*-acting elements to be about 30 nt mainly upstream of the editing site. Plant site-specific editing factors belong to the PLS subfamily of pentatricopeptide repeat (PPR) protein family. PPR proteins are defined by tandem arrays of a degenerate 35 amino acid motif, the PPR motif [9]. The PLS subfamily is characterized by the presence of shorter (S) and longer (L) repeats than the canonical motif of 35 aa (P); this family can be further separated into smaller subclasses based on a series of characteristic C-terminal domains, the E and DYW domains [9]. The recognition code between the PPR proteins and their RNA targets has recently been identified [10].

RIP1, a protein that interacts *in vivo* with RARE1, a PPR-DYW plastid protein that controls the editing of *accD*-794 [11], [12], has been shown to be dual-targeted to chloroplasts and mitochondria and to control editing in both organelles. In particular, 266 mitochondrial editing events were found to have reduced efficiency in a *rip1* mutant, with major loss of editing at 108 C targets [12]. *RIP1* belongs to a 10-member gene family; some of its members were also reported to encode plant editing factors by another group, who named the gene family Multiple Organellar RNA editing Factors (MORFs) [13] (Table 1).

**Table 1:** Description of the genes, protein product locations, and mutants used in this study.

*RIP* id^1^	*MORF* id^2^	gene id	location^3^			VIGS	T-DNA mutant			
			MS/MS	Predotar	TargetP			*rip* #	*morf #*	genetic background
*RIP1*	*MORF8*	At3g15000	M, P	P	P	NO	FLAG_150D11	*rip1-1*		WS
*RIP2*	*MORF2*	At2g33420	plastid	P	M	YES				
*RIP3*	*MORF3*	At3g06790				NO	GK-109E12.01	*rip3-1*	*morf3-1*	Columbia
							SAIL_156_A04	*rip3-2*		Columbia
*RIP4*	*MORF4*	At5g44780		M	M	NO	SAIL_731_D08	*rip4-1*	*morf4-1*	Columbia
							SALK_114438	*rip4-2*		Columbia
*RIP5*	*MORF5*	At1g32580	M	M	P	YES	SALK_016801	*rip5-1*		Columbia
*RIP6*	*MORF6*	At2g35240		M	P	NO	GK-184F04.01	*rip6-1*	*morf6-1*	Columbia
*RIP7*	*MORF7*	At1g72530		M	M	YES				
*RIP8*	*MORF1*	At4g20020		M	M	YES				
*RIP9*	*MORF9*	At1g11430	plastid	P	P	YES				
*RIP10*		At1g53260		P	P	YES				

1 Since two nomenclatures have been given to this family, *RIP* or *MORF*, we propose to use the same index for each gene, e.g. *MORF2* = *RIP2*, except for *RIP1* which has been described as *MORF8*, and *MORF1* which should then be referred as *RIP8*.

2 from Takenaka et al. (2012).

3 location retrieved from the Subcellular Proteomic Database [30]. MS: mass spectrometry M: mitochondrion, P: plastid.

Wild-type plants and plants with mutations in PPR protein-encoding genes or *RIP* family members have been assayed for editing extent of Cs in mitochondrial and chloroplasts by such methods as bulk Sanger sequencing of RT-PCR products, sequencing of individual cDNA clones, high-resolution melting (HRM) analysis of cDNA amplicons, single-nucleotide extension polymorphism typing, and poisoned primer extension (PPE) assays [2], [14]–[17]. All these methods suffer from either specific and/or general limitations. When adapted to screening many sites, some lack sensitivity and accuracy (bulk-sequencing of RT-PCR amplicons), or are resource- and time-consuming (e.g., single-nucleotide extension, which necessitates the use of specific primers for each editing site). HRM cannot detect the number of editing sites in an amplicon nor their location. The PPE assay, which is truly quantitative, is too labor-intensive and prohibitively expensive for large-scale surveys.

The advent of high-throughput sequencing technologies has permitted the direct sequencing of cDNA generated from messenger and structural RNAs (RNA-seq) at a genomic scale [18]–[20]. Here we report the use of Illumina sequencing of plant organelle cDNAs to quantify editing extent in mutant and silenced genes of the Arabidopsis *RIP* family. Unlike three previous reports on the use of RNA-seq to study organelle RNA editing [21]–[23], our analysis focuses on organelle transcripts corresponding to known genes, simplifying the bioinformatic analysis and increasing the depth coverage. We describe here a complete characterization of the editing phenotypes that result from mutating or silencing every member of the RIP editing factor family.

## Results

### Sequencing of organelle RT-PCR products from leaf tissue of plants with altered expression of *RIP* family members

**Figure 1:**
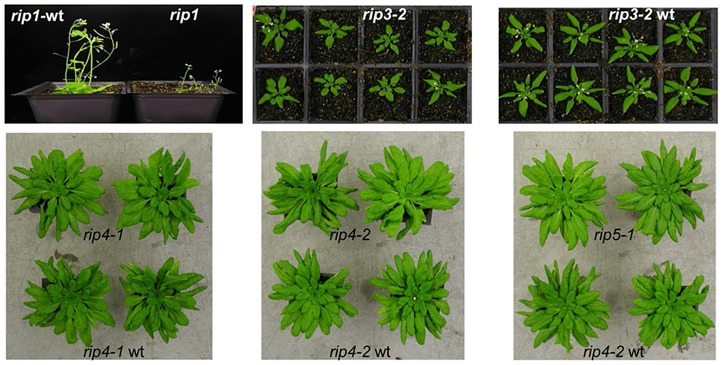
The *rip1* mutant is the only mutant in our study with a severe defective phenotype. *rip1*, *rip1*-wt, *rip3-2* and *rip3-2*-wt were grown under long-day growth room conditions (14 h light/10 h dark) while the other plants were grown in short day conditions (10 k light/14 h dark). *rip3-2* mutant plants show a slight delay in development compared to their wild-type siblings. At the time of photography, 2 out of 8 (1/4) *rip3-2* seedlings (30 days post-sowing) have flowered while all the wild-type seedlings have flowered (white spots in the middle of each plant are the flowers). *rip4-1*, *rip4-2*, and *rip5-1* do not show any phenotypic difference with their wild-type (a *rip5-1* wild-type was not available for the picture, but its genetic background is Columbia like *rip4-2*).

In order to evaluate the role of all 10 Arabidopsis *RIP* family members in editing of chloroplast and mitochondrial transcripts, we obtained mutants in 5 RIP family members from several different stock centers and performed Virus-Induced Gene Silencing (VIGS) for RIP family members for which mutants were not available (Table 1). The *rip1* mutant has a dwarf phenotype and the *rip3* mutant exhibits a slight delay in development; the other mutants do not exhibit any phenotypic defect under growth room conditions (Figure 1). RNA was extracted from leaves of the mutants, wild-type or silenced plants, reverse-transcribed, and PCR was performed with primers that amplify all transcripts encoding either mitochondrial or chloroplast genes (Table S1). Nineteen plastid RT-PCR products were amplified that encompass known editing sites [2]; 34 mitochondrial known genes or ORFs were reverse transcribed because they were reported to contain editing sites [24]. *Cox1* is the only mitochondrial gene not covered by our analysis because of its reported lack of editing sites [24]. We included 7 mitochondrial RT-PCR products that cover untranslated regions and contain reported editing sites [24] (Table S1). The RT-PCR amplicons were quantified and mixed in equimolar ratio for each plant. The cDNA mix was then sheared by ultra-sonication and used as a template for the production of an Illumina TruSeq RNA library. We refer to our method as strand-and transcript-specific RT-PCRseq (STS-PCRseq). Thirty libraries with different indexes, obtained from 7 mutants, 6 wild-types, 6 silenced, and two controls for the VIGS experiment were obtained (Table 2). One of the wt cDNA mixes was used twice to produce libraries with different indexes to test for the reproducibility of the measure of editing extent by RNA-seq (Table 2, see below).

**Table 2:** Statistics of number of reads/editing site for the 30 Illumina libraries analyzed in this study.

cDNA	Library	average	sd	min	max	number of libraries pooled
*rip1*	s14	7773	7325	505	55568	24
*rip1wt*	s15	4388	4411	227	37813	24
*rip3-1*	s16	6840	5863	359	40565	24
*rip3-1wt*	s18	8154	8784	374	79326	24
*rip5-1*	s20	6754	8969	192	61229	24
*rip6-1*	s21	9082	10485	373	64530	24
*rip3-2*	s22	6388	8672	201	59421	24
*rip4-1*	s23	10917	16168	395	111752	24
*rip4-2*	s25	7597	10115	270	69687	24
*rip9*-silenced	a1	1503	2959	35	36769	24
*rip8*-silenced	a2	4294	5682	108	53537	24
GFP-silenced	a3	2796	2912	95	24460	24
Col-GFP	a4	3222	6113	108	80994	24
*rip2*-silenced	a5	3822	4332	95	40009	24
*rip5*-silenced	a6	5144	9673	143	124100	24
*rip10*-silenced	a7	4097	5825	62	58476	24
*rip7*-silenced	a8	550	494	11	4653	24
*rip5-1wt*	a9	4192	3683	126	27909	24
*rip3-2wt*	a10	4558	4330	180	37560	24
*rip4-1wt*	a11	1914	1709	73	12882	24
*rip4-2wt*	a12	4250	3796	171	35858	24
*rip3-1wt*	a13	13795	12477	410	90356	24
*rip3-1*	L7	14928	21543	136	199680	12
*rip3-1wt*	L12	12383	19494	213	186895	12
*rip4-1*	L13	13807	22808	5	199739	12
*rip4-1wt*	L14	55639	53291	883	200025	12
*rip4-2*	L15	15417	23628	245	199998	12
*rip4-2wt*	L16	11811	18260	147	166682	12
*rip6-1*	L18	12709	19921	117	189667	12
Col-GFP	L19	12557	17212	204	161254	12
	total	9043	18095	5	200025	

s18 and a13 were obtained with the same cDNA.

Biological replicates for some mutant and wild-type were analyzed to test for the variability of editing extent in our study. These biological replicates are libraries obtained from different plants of the same genotype grown at the same time and in the same conditions (Table 2). Unlike other libraries in this study, the libraries from biological replicates (L samples in Table 2 and Dataset S1) were sequenced in a pool of 12 libraries while other pools contained 24 libraries.

### STS-DNA-seq identifies 656 organelle editing sites, among which 133 are new sites

The depth of the reads was surveyed for each library every 10 bp along each gene. A characteristic pattern is shown in Figure S1 for *rip1*. Amplicons whose size is smaller than the shearing cutoff (300 bp) showed a flat pattern of number of reads along the template (Figure S1A); amplicons whose size is larger than 300 bp exhibit either a drop in the number of reads in the middle of the template (Figure S1B) or a peak at the 5′ or the 3′ end of the template (Figures S1C, and D respectively). The drop in reads in the middle of the template can be explained by the non-randomness of the template shearing, which could generate more fragments with extremities if the break occurs only once inside the template. The reason for uneven distribution of reads at the 5′ or 3′ end of the template is unknown.

For each sample, we scanned all the 8320 C sites on the genomic templates to identify sites where the number of T bases in aligned reads was found to be statistically significant. The statistical significance was assessed using a likelihood ratio test comparing how well the observed alignment can be explained by assuming the absence of an edit and assuming an edit with certain proportion of T. The test used empirical mismatch rates calculated for each library from alignments. Details on this calculation and on the statistical test itself are given in protocol S1. Using this procedure, we identified 1833 sites where p-value was less than 1e-6 in at least one sample. These putative editing sites were filtered further by combining two additional criteria: the editing fraction (T/C+T) had to be larger than 0.05 in at least one sample and the average number of reads across all the samples had to be larger than 100.

DNA sequencing runs at the Cornell facility always contain controls to verify that the error rate is acceptably low. In addition to these routine controls, we spiked some of the samples with DNAs obtained from a plasmid preparation, a PCR amplification, and a RT-PCR amplification to empirically estimate the mismatch rate that occurs when each type of template undergoes sequencing. This empirical estimate, described in detail in Protocol S1 (section “Estimating mismatch rates”), involves aligning reads to their respective templates and collecting mismatch counts as functions of position on the read. As such, the empirical rates account for errors introduced at the experimental stage, detectable by the applied read alignment strategy. A representative result is given in Figure S2. The mismatch rates are in the order of 1e-4 for the plasmid preparation template; different mismatch rates between different pairs of bases are quite similar to each other (Figure S2A). Alignments to PCR (DNA) and RT-PCR (cDNA) spikes and to the whole genome (organelle genomic templates) give very similar ordering and values of mismatch rates (Figure S2B, C, and D). Except for the elevated A->G rate, these patterns are different from those seen for the plasmid preparation template (Figure S2A), and the values are 5–10 times higher, probably due to the errors introduced by the polymerases. The C->T mismatch rate derived from alignments to the whole genome (Figure S2D) is higher than in other cases since it contains contributions from some low-level CT editing sites which were not masked when the mismatch counts were collected (only the most obvious editing sites were masked). This elevated C->T mismatch rate makes our calls of CT editing sites more conservative. Overall, the total mismatch rate derived from PCR, RT-PCR and whole genome is quite low, approximately 1e-3 along the read.

We identified 656 editing sites in the filtered data, among which 37 are plastid and 619 are mitochondrial (Dataset S1). Five hundred and twenty-three of the editing sites identified in this analysis have been previously reported [2], [3], [13], [21], [24] (Dataset S2). These previously reported sites have a rather high average editing extent of 0.81 among the wild-type accessions. The proportion of silent sites, those which do not change the encoded amino acid, is low when compared to the non-silent sites, 17% vs. 83%, respectively (Dataset S2). In addition the average editing extent of silent sites is smaller than the average editing extent of non-silent sites, 0.44 vs. 0.89, respectively.

**Figure 2:**
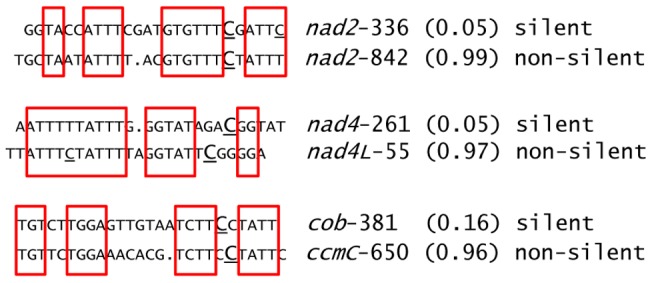
Examples of newly identified editing sites having sequences in their putative *cis* elements similar to known editing sites. The sequences shown are 20 nt upstream and 5 nt downstream around the target C for editing (underlined and capitalized). Other Cs that are edited are underlined. The upper (lower) sequence belongs to a new (known) editing site. The names of the sites are given on the right of each sequence with the average editing extent found in the wild-type in between parentheses. Identical sequences are highlighted by red squares. Upon visual inspection, gaps were introduced in order to increase the similarity between *cis* elements.

The average editing extent among the wild-type accessions of the 133 new editing sites identified in this study is only 0.07, which is markedly smaller than for the reported sites (Dataset S3). The proportion of silent sites in this population is higher than in the previously reported sites, 63% vs. 17%, respectively. Among the newly detected edited sites, the ranges and averages of editing extent among wild-type accessions between silent and non-silent sites are quite similar, 0.008–0.24 vs. 0.005–0.23 and 0.07 vs. 0.05, respectively (Dataset S3). The low-level editing we have detected might be considered “accidental” editing due to similarity of nearby sequences to *cis*-elements that are present upstream of other, more highly edited C targets. We looked for similarity of sequences surrounding these new sites (−20 +5) with previously reported sites. Thirty four new editing sites exhibit similarity (≥10 nt) in their putative *cis* sequences to reported sites (Dataset S3). Examples of similar putative *cis* elements between three new editing sites and some reported sites is given in Figure 2. We also examined the lists of editing sites in three other species, *Lotus japonicus*, rice and tobacco, to determine whether any of the new Arabidopsis C targets had been identified in other well-characterized organelle transcriptomes. 8, 5 and 1 of these new sites have been reported in tobacco, rice, and Lotus, respectively (Dataset S3). Overall, 12 of these new Arabidopsis editing sites are also present in the other species that have been analyzed.

**Figure 3:**
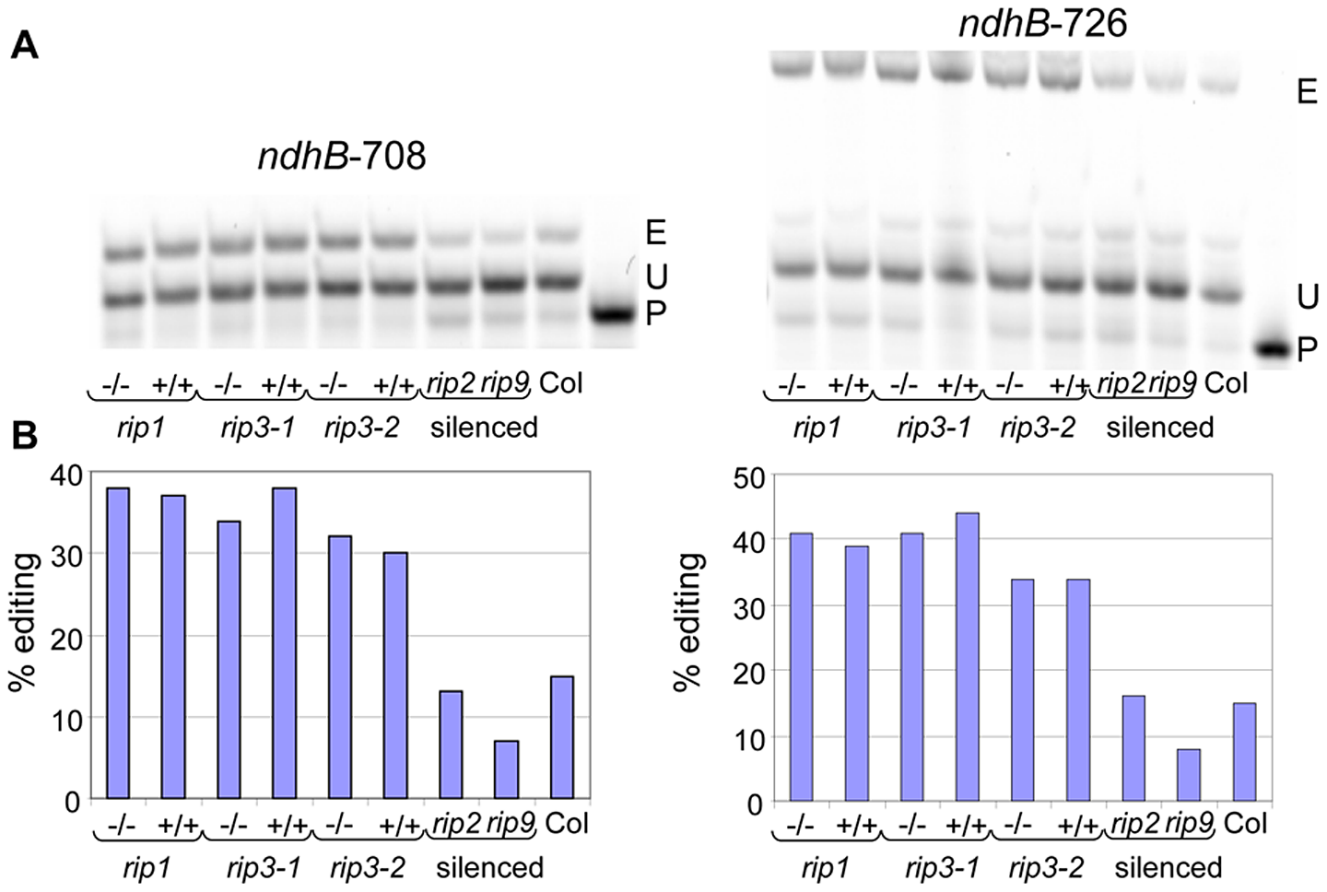
Validation by PPE of two plastid sites detected by RNA-seq. (A) Acrylamide gels separate the PPE products obtained from some samples used in this study; −/−, T-DNA mutant; +/+, wild-type. E, edited; P, primer; U, unedited. The name of the site assayed is given above each gel. (B) The quantification of editing extent derived from the measure of the band's intensity is represented by a bar below each lane of the acrylamide gels.

Three new plastid editing sites were also identified in this study; they all lie on the *ndhB* transcript at position 153, 708 and 726 (Dataset S2). We assayed two of these new sites, *ndhB*-708 and *ndhB*-726, by poisoned primer extension (PPE). The PPE analysis confirmed the existence of editing at these locations and validated the RNA-seq finding that the *rip1* and *rip3* mutants and their wild-type siblings exhibited higher editing extent at these two sites than did the Columbia GFP-expressing transgenic line that was used in the VIGS experiments, which exhibited only 15% editing (Figure 3).

The sensitivity of deep sequencing over conventional methods such as RT-PCR bulk sequencing to detect editing is supported by the finding of numerous new sites. A T peak (which should be present in the edited faction) in sequencing electrophoretograms is often absent at sites whose editing extent is assayed at 10% by deep sequencing (Figure S3). Thus, the threshold of editing extent detection by bulk sequencing can be estimated to be around 10% and this low level of sensitivity explains why the new sites, whose average editing extent is 7%, were not detected before the use of deep sequencing.

### Validation of the RNA-seq method to assess organelle RNA editing

**Figure 4:**
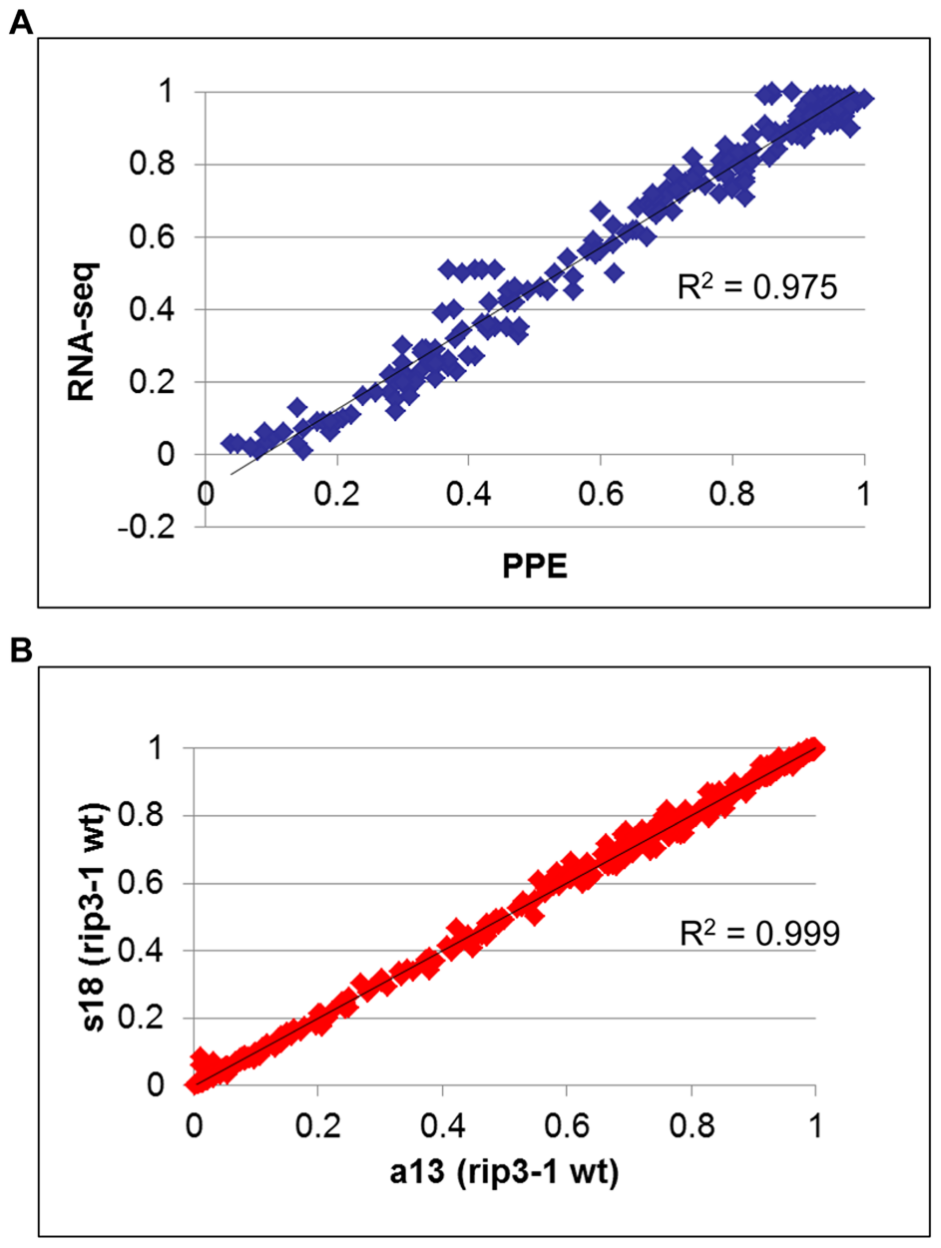
Measurement of RNA editing extent by RNA-seq is accurate and highly reproducible. (A) The editing extent of 226 organelle editing events, 106 mitochondrial :9 sites and 3 to 19 genotypes assayed per site and 120 plastid: 12 sites and 7 to 11 genotypes assayed per site, was measured by RNA-seq and PPE assay. A high correlation with PPE assay (R^2^ = 0.97), the most precise method to measure editing extent, demonstrates the robustness of RNA-seq to evaluate organelle editing extent. (B) Two libraries with different indexes were prepared from the same sheared cDNA obtained from *rip1* mutant. The editing extent measured between the two libraries shows a very high correlation (R^2^>0.99).

In order to assess the reliability of the measure of editing extent by deep sequencing, we performed 226 PPE assays on either mitochondrial or plastid C targets. The correlation between the PPE assay, which is known to be the most robust measurement of editing extent and deep sequencing, was found to be >0.95 (Figure 4A). The slight discrepancy between PPE and deep sequencing concerns the absolute value of editing extent, which tends to be smaller when assayed by Illumina sequencing (Figure 4A). The reproducibility of editing extent measurement by deep sequencing was tested by using the same sheared cDNA to construct two libraries with different indexes, s18 and a13 respectively (Figure 4B). The correlation between these two experiments is >0.99, demonstrating the reproducibility of editing extent measurement by deep sequencing of RT-PCR products.

**Figure 5:**
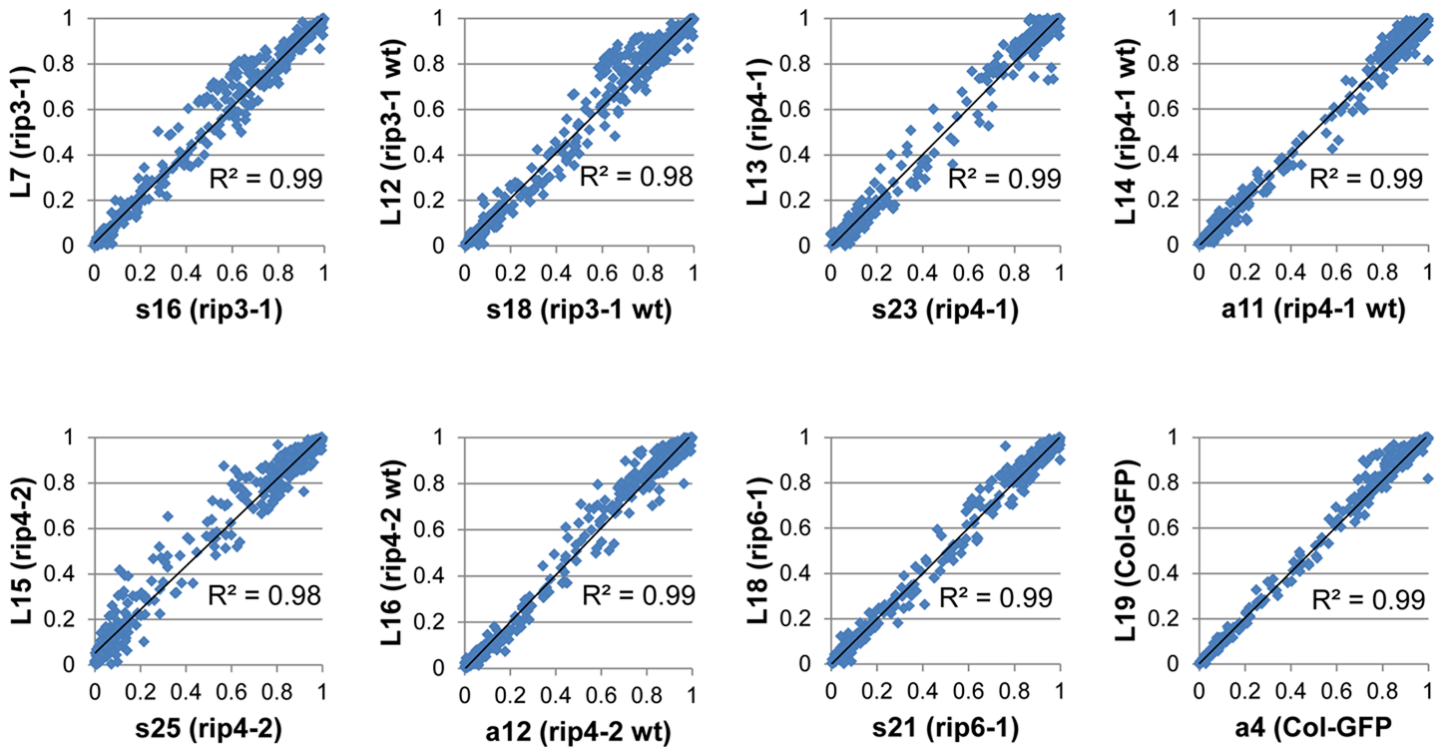
Biological replicates exhibit a high correlation of editing extent. Each graph represents a pairwise comparison of editing extent that was measured on two libraries obtained from cDNAs of two plants belonging to the same genotype grown in the same conditions and harvested at the same time.

We included in our analysis 8 biological replicates corresponding to 4 *rip* mutants and 4 wild-type accessions. Libraries were made from cDNA obtained from different plants grown in the same conditions (Table 2). The variability of editing extent between these biological replicates is negligible as demonstrated by the very high correlation found for each pairwise comparison (Figure 5).

### Analysis of a *rip1* mutant by STS-PCRseq confirms *RIP1* to be a major editing factor and validates the new editing sites

In mitochondria of a *rip1* T-DNA insertional mutant, 368 sites were previously surveyed by bulk sequencing; 108 of these sites or *ca* 29% were found to exhibit a pronounced reduction of editing extent, while 72% showed a detectable decrease of editing extent in the bulk sequencing electrophoretograms [12]. We now report the characterization of this mutant's organelle RNA editing extent by deep sequencing. The quantitative nature of the Illumina sequencing assay allowed us to measure the effect of RIP1 on the editing extent of a particular site according to the variation of editing extent (ΔEE) observed in the *rip1* mutant relative to the wt: ΔEE = (wt-*rip1*)/wt. We tested by a chi-square test for the significance of a difference in the editing fraction in *rip1* mutant vs. wild-type. We adopted the Bonferroni correction to account for repetitive testing, which is a simple way to control the familywise error rate (α) by simply dividing it by the number of comparisons (n) to derive the nominal threshold for each test (β = α/n). In the case of mitochondrial sites, comparisons of 619 sites were made between the *rip1* mutant and its wild-type sibling; therefore, a nominal threshold of 1.6e-6 was chosen in order to achieve an overall significance of 1e-3. We classified the sites into two categories depending on the result of the chi-square test and the value of ΔEE. If the site exhibited P (chisq) >1.6 e-6, we included this site in the *rip1*-independent class. Otherwise, the site was classified as *rip1*-independent if ΔEE<0.1 or *rip1*-dependent if ΔEE≥0.1. Overall, the percentages of *rip1*-dependent sites are very similar between the bulk-sequencing and the deep-sequencing studies, 72% vs. 77% respectively ([12] and Table 3). The deep-sequencing data confirmed the biased distribution of *rip1*-dependent sites according to the mitochondrial complex to which the site belongs. For instance, there is a marked excess of *rip1*-dependent sites in cytochrome c biogenesis and ribosomal transcripts, 90% and 89%, respectively (Table 3). On the other hand, the excess of *rip1*-dependent sites is less pronounced in the complex I transcripts (57%, Table 3). The *mttb* transcript carries by far the highest proportion of *rip1*-dependent sites (98%, Table 3).

**Table 3:** Number and percentage of *RIP1*-independent (Δ<0.1) and *RIP*-dependent (0.1≤Δ) mitochondrial sites.

	Reported sites			New sites			All sites (reported + new)		
	Δ<0.1	0.1≤Δ	total	Δ<0.1	0.1≤Δ	total	Δ<0.1	0.1≤Δ	total
complex I	87	107	194	14	26	40	101	133	234
complex I%	45	55		35	65		43	57	
*cob* -complex III	3	7	10	1	3	4	4	10	14
*cob* -complex III%	30	70		25	75		29	71	
complex IV	5	20	25	1	4	5	6	24	30
complex IV%	20	80		20	80		20	80	
complex V	1	19	20	1	6	7	2	25	27
complex V%	5	95		14	86		7	93	
cytochrome c biogenesis	8	116	123	8	26	34	16	142	157
cytochrome c biogenesis%	7	94		24	76		10	90	
ribosomal protein	2	65	67	8	13	21	10	78	88
ribosomal protein%	3	97		38	62		11	89	
*matR*	0	10	10	2	4	6	2	14	16
*matR%*	0	100		33	67		13	88	
*mttb (OrfX)*	0	34	34	1	9	10	1	43	44
*mttb (OrfX)%*	0	100		10	90		2	98	
TOTAL	106	377	483	36	91	127	142	468	610
TOTAL%	22	78		28	72		23	77	

Splitting the analysis of *rip1*-dependent and independent mitochondrial sites between the reported and the new sites shows a very similar effect of the *rip1* mutation on both types of sites (Table 3). The proportion of sites affected by the *rip1* mutation is not statistically different in the reported sites compared to the new sites, 78% vs. 72%, respectively (Table 3, P = 0.13). Moreover, the distribution of *RIP1*-dependent and independent classes in the mitochondrial complexes shows a good agreement between the reported sites and the new sites (Table 3).

The data confirmed *RIP1* as a major component of the plant editing machinery: 474 mitochondrial sites or 77% and 8 plastid sites or 22% showed a ΔEE>0.1 (Tables 4 and 5). Among the plastid sites, *petL*-5 showed the most pronounced reduction of editing extent in the *rip1* mutant (ΔEE = 0.81, Table S2). Unlike the mitochondrial editing sites, comparison of the variation between the Illumina-sequencing assay of chloroplast transcript editing extent in the *rip1* mutant to the previous results obtained by PPE exhibited some discrepancies between the two studies (Table S2). Four sites, *petL*-5, *accD*-794, *rpl23*-89, and *ndhD*-878, exhibited lower editing extent in the current study, while the *rps12*-intron, and *rpoC1*-488, had a consistent increase of editing extent relative to the prior PPE findings (Table S2). On the other hand, sites in the *rpoB* transcript and *ndhF*-290 exhibited lower extent by STC-PCRseq, while they were invariant or had an increase of editing extent when assayed by PPE. Given that we have proven that deep sequencing is a reliable way to measure editing extent, the discrepancy between the two studies is likely to be linked to the RNA samples, which were taken from two different sets of plants for use in the plastid editing assays. We therefore performed PPE on the new RNA samples that were used in our Illumina sequencing study. When 8 of the C targets that exhibited the greatest variation between the two studies were assayed by PPE, there was an increase in the correlation of the PPE assay and the deep sequencing from 0.55 to 0.82 and 0.62 to 0.81 for the *rip1* mutant and wild-type, respectively (Figure S4), indicating that a difference in editing extent exists in the RNA samples, likely due to environmental factors and/or developmental stage.

**Table 4:** Number (percentage) of *RIP*-dependent (ΔEE≥0.1) and *RIP*-independent (ΔEE<0.1) mitochondrial sites in the *RIP* mutant and *RIP*-silenced plants.

RIP	ΔEE≥0.1	0.1>ΔEE
*rip1*	474 (77)	145 (23)
*rip3-1*	183 (30)	436 (70)
*rip3-2*	201 (32)	418 (68)
*rip4-1*	19 (3)	600 (97)
*rip4-2*	6 (1)	613 (99)
*rip5-1*	25 (4)	594 (96)
*rip5*-sil	12 (2)	574 (98)
*rip7*-sil	6 (1)	580 (99)
*rip8*-*sil*	74 (13)	512 (87)
*rip2*-*sil*	21 (4)	565 (96)
*rip9*-*sil*	1 (0.2)	585 (99.8)
*rip10*-*sil*	0 (0)	586 (100)

619 (586) sites were analyzed in the mutant (silenced) plants.

**Table 5:** Number (percentage) of *RIP*-dependent (ΔEE≥0.1) and *RIP*-independent (ΔEE<0.1) plastid sites in the *RIP* mutant and *RIP*-silenced plants.

RIP	ΔEE≥0.1	0.1>ΔEE
*rip1*	8 (22)	29 (78)
*rip3-1*	2 (5)	35 (95)
*rip3-2*	7 (19)	30 (81)
*rip4-1*	0 (0)	37 (100)
*rip4-2*	0 (0)	37 (100)
*rip5-1*	0 (0)	37 (100)
*rip5*-sil	0 (0)	36 (100)
*rip7*-sil	0 (0)	36 (100)
*rip8-sil*	0 (0)	36 (100)
*rip2-sil*	22 (61)	14 (39)
*rip9-sil*	24 (67)	12 (33)
*rip10-sil*	1 (3)	35 (97)

37 (36) sites were analyzed in the mutant (silenced) plants.

### The majority of sites controlled by *RIP3 and RIP8*, two moderately important mitochondrial factors, overlap with the sites under *RIP1* control

We studied two *rip3* T-DNA mutants (GK-109E12.01 and SAIL_156_A04) and their wild-type siblings by STS-PCRseq (Table 1). *RIP3* can be considered a moderately important mitochondrial editing factor because of the number of sites it controls. There are 183 sites that exhibit a ΔEE≥0.1 in *rip3-1* and 201 in *rip3-2*, while the *rip1* mutant has 474 sites with altered editing extent (Table 4). We investigated what proportion of the sites was controlled by *RIP1* alone, *RIP3* alone, both of them, or neither of them (Table S3). A chi-square test demonstrates that the effects of *RIP1* and *RIP3* on mitochondrial editing extent cannot be considered statistically independent (P<10^−4^). There is an excess of observed sites controlled by both factors (ΔEE≥0.1), and an excess of observed sites not controlled by any factor (ΔEE<0.1) compared to the number of sites expected in case of independence (Table S3). Therefore, our data indicate a synergistic effect of these two factors on mitochondrial editing extent. The survey of plastid sites in both *rip3* mutants indicates that some sites are significantly reduced in editing extent, especially in *rip3-2* (Table 5); however these sites are not common between the two mutants and thus can be dismissed as not caused by the mutation. The reason for these sites being specifically reduced in one mutant but not the other are not clear but *rpoC1*-488, the site showing the most reduced editing extent in *rip3-2* is the most variable site in other *rip* mutants, where it shows an increase of editing extent compared to the wild-type (Table S2). Unlike the plastid sites, the vast majority of mitochondrial sites, *ca* 90% (163/183) that are significantly decreased in editing extent in the *rip3-1* mutant exhibit also a significant decrease of editing extent in the *rip3-2* mutant.

The T-DNA insertional mutant we obtained in *RIP8* is embryo lethal in the homozygous state. We therefore used VIGS to knock down the expression of this gene. Because VIGS in Arabidopsis is sometimes inefficient, we silence Arabidopsis plants carrying a GFP gene, and screen for leaf tissue on silenced plants that exhibit loss of GFP fluorescence, as the gene of interest is then also found to be silenced. Two types of control plants were used in this experiment: uninoculated plants and plants inoculated with a silencing vector containing only GFP (libraries a4 and a3, respectively, Table 2). 33 mitochondrial sites and 1 plastid site exhibited a significant reduction of editing extent in the GFP-silenced control when compared to the uninoculated plants and were discarded in the analysis of silenced plants. Sites significantly reduced in the *rip8*-silenced plants vs. uninoculated plants were further checked against the GFP-silenced control.

Like *RIP3*, *RIP8* can be considered a moderately important mitochondrial editing factor; 74 sites exhibit a ΔEE≥0.1 in *RIP8* silenced plants (Table 4). The number of affected sites is likely to be underestimated because silencing does not completely eliminate expression. Our data indicate that the effect of RIP1 and RIP8 on mitochondrial editing is not independent, although the deviation from independence is less pronounced for them than for RIP3 and RIP8 (P<0.05 and P<0.01, respectively, Table S4). Like for the pair (RIP1, RIP3), each pair of mitochondrial factors, (RIP1, RIP8) or (RIP3, RIP8) shows an excess of observed sites controlled by both factors (ΔEE≥0.1), and an excess of observed sites not controlled by any factor (ΔEE<0.1) compared to the number of sites expected in case of independence (Table S4).

**Figure 6:**
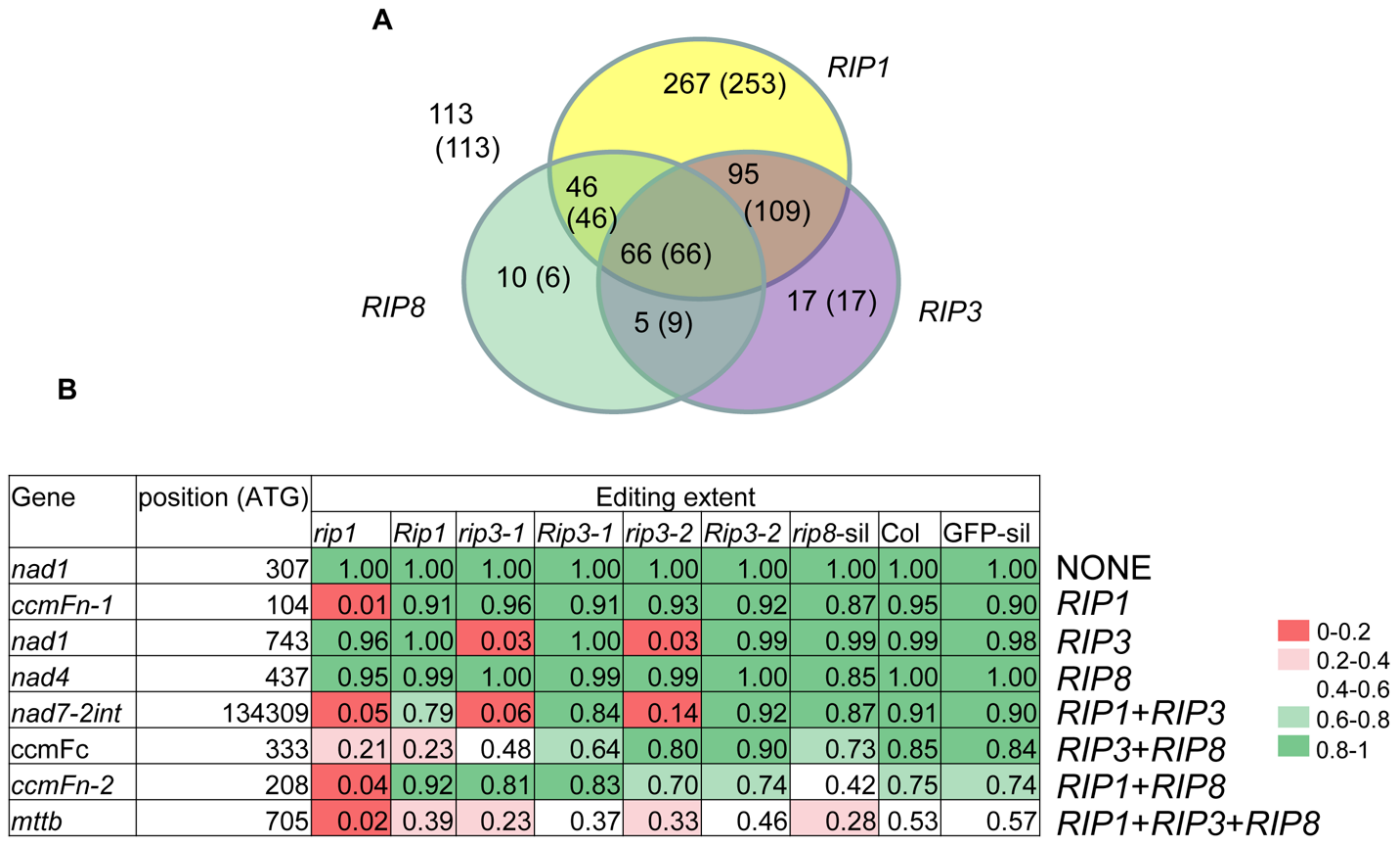
Relative importance of RIPs on mitochondrial editing. (A) Number of mitochondrial sites under the control of RIPs (ΔEE≥0.1, P<1.6 e-6). *RIP8* results were obtained from VIGS (all the mitochondrial sites were counted in this analysis). Numbers of sites for *RIP3* refer to *rip3-1* (*rip3-2*). (B) Examples of mitochondrial sites falling into one of the eight categories described in the Venn diagram shown in (A). The background color reflects the range of editing extent from red (low: 0–0.2) to dark green (high: 0.8–1).

Analysis of the overlapping effects of *RIP1*, *RIP3* and *RIP8* on mitochondrial editing demonstrates that the majority of sites controlled by *RIP3* and *RIP8* are also controlled by *RIP1* (Figure 6). The proportion of sites controlled either by *RIP8* or *RIP3* that are also controlled by *RIP1* is remarkably similar, 112/127 or 88% and 161/183 (175/201) or 88% (87%), respectively (number in parenthesis refer to *rip3-2*) (Figure 6). Sites controlled by only one factor are predominantly found in *RIP1*-controlled sites whereas they represent only a small fraction in *RIP3* and *RIP8*-controlled sites (Figure 6).

### A majority of plastid sites controlled by *RIP2* are also under the control of *RIP9* and *vice versa*

RIP2 and RIP9 are both located in plastids. RIP2 was shown to be imported *in vitro* into chloroplasts [25] and RIP9 has been found in the stromal proteome of *Arabidopsis thaliana* chloroplasts [26]. We investigated the role of the *RIP2* and *RIP9* genes in plastid editing by silencing them and comparing the editing extent in silenced plants vs. control plants. 22 plastid sites among 36 surveyed (61%), show a reduction in editing extent ≥0.1 in *RIP2*-silenced plants (Tables 5 and S5). We also observed that 21 mitochondrial editing sites exhibit a reduction in editing extent ≥0.1 in *RIP2*-silenced plants (Tables 4 and S6).

In *RIP9*-silenced plants, 24 (67%) plastid sites exhibit reduced editing extent ≥0.1 when compared to the uninoculated control plants (Tables 5 and S7). We also detected a reduction in editing of 1 mitochondrial site (ΔEE≥0.1) in the *RIP9*-silenced plants (Table 4).

**Figure 7:**
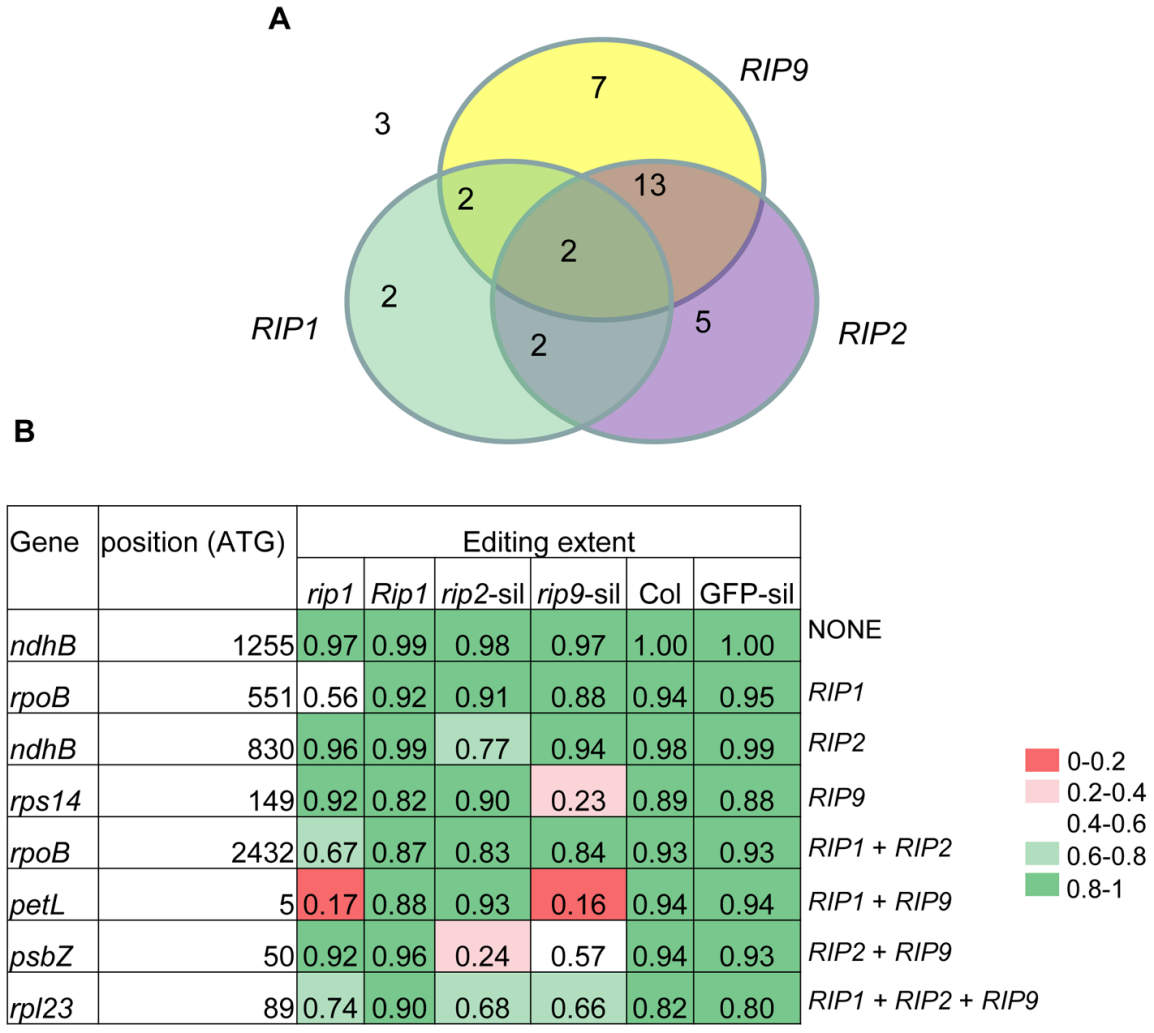
Relative importance of RIPs on plastid editing. (A) Number of plastid sites under the control of RIPs (ΔEE≥0.1, P 2.7<10^−5^). *RIP2* and *RIP9* results were obtained from VIGS. (B) Examples of plastid sites falling into one of the eight categories described in the Venn diagram shown in (A). The background color reflects the range of editing extent from red (low: 0–0.2) to dark green (high: 0.8–1).

The effects of *RIP2* and *RIP9* on plastid editing extent are statistically independent (Table S8). However, *RIP2* and *RIP9* overlap in their control of plastid editing extent; 15 sites are under the control of both *RIP2* and *RIP9* (Figure 7). The proportion of sites controlled by only one plastid factor are 25% (2/8), 23% (5/22), and 29% (7/24) for *RIP1*, *RIP2*, and *RIP9*, respectively.

### Other members of the RIP family exhibit only minor effects on RNA editing in mutants and silenced plants

We analyzed the remaining members of the *RIP* family, *RIP4*, *RIP5*, *RIP6*, *RIP7*, and RIP10 (Table 1). For *RIP4*, we studied two T-DNA insertional mutants, *rip4-1* and *rip4-2*. *RIP5* was analyzed both in a T-DNA insertional mutant, *rip5-1* and in VIGS plants. In our comparison of editing between mutant and wild-type, we compared the wild-type sibling to the mutant, except for *RIP6*, for which we had data only for the mutant. *RIP7* and *RIP10* were analyzed only in plants that had undergone VIGS.

*RIP4*, *RIP5*, *RIP6*, *RIP7*, and *RIP10* affect a small number of mitochondrial editing sites and none of the plastid sites except for one plastid site affected in *RIP10* silenced leaves (Tables 4 and 5). *CcmFc*-1150 is the only site that was found to have reduced editing extent in both *rip4* mutants. Among the 12 sites that show a significant reduction of editing extent (ΔEE≥0.1) in *rip5*-silenced plants, two sites also exhibit a significant reduction in *rip5* mutant plants (Table S9). Among the 6 editing sites that exhibit a significant reduction of editing extent (ΔEE≥0.1) in *rip7*-silenced plants, half are on the *nad3* transcript (Table S10).

Because *rip6* wild-type sibling data was not available, in order to establish the basis for comparison of the *rip6* mutant plant's editing extent to wild-type, we estimated the wild-type level by averaging the editing extent found in the wild-type siblings of *rip3*, *rip4*, and *rip5* plants and the Columbia accession used for VIGS. Because approximately 80% of the mitochondrial sites show a significant difference of editing extent among Columbia accessions (P(χ2)<10^−6^), we did not consider these sites in our *rip6* mutant analysis. We did not find any site with a significant reduction in *rip6* plants (data not shown),

Some of the mutants analyzed in this study have been analyzed by bulksequencing [13]. Our results are in very good agreement with those previous data; for instance all the sites significantly reduced in *rip3-1* mutant and that show a difference in editing with the wild-type >0.25 were also detected by bulksequencing [13]. There are, however, some slight differences between the two studies, which are summarized in Table S11.

## Discussion

### STS-PCRseq : a new method to measure organelle editing extent

We have developed a new method to study plant organelle editing and demonstrate its value in characterizing an Arabidopsis gene family encoding organelle-targeted proteins. The method, based on Illumina sequencing, is sensitive, quantitative, and robust. It combines the simplicity of RT-PCR bulk sequencing for screening many sites simultaneously along with the accuracy and sensitivity of the PPE assay. Our new approach is also more economical. Currently 48 libraries can be produced from one Illumina kit, resulting in a cost per library of about $63. In our facility, the cost of 100 nt reads per library is $50 when multiplexing 24 libraries. Therefore, the cost in materials for producing and facility use for sequencing a sample is about $113; given that we analyzed 656 editing sites, the cost/editing site in our study was around 17 cents/site, or 220 times cheaper than bulk sequencing of RT-PCR products. Illumina sequencing of RT-PCR products is about 30 times cheaper than HRM, which was estimated to be 6 times less expensive than bulk sequencing [2].

Current methods for assaying plant organelle editing extent, such as single-base extension, localized RT-PCR bulk sequencing, and PPE require a prior knowledge of existing editing sites [16]. By using primers that result in amplification of cDNAs corresponding to most of the organelle open reading frames, prior knowledge of the location of C targets is not necessary. Judicious selection of primers could also allow assay of splicing as well as editing. A higher level of multiplexing than we used would be possible while still maintaining a sufficient read number, as the average depth/editing site we obtained was about 5500 when pooling 24 libraries (Table 2). The limiting factor at the time of this study was the number of different commercially available indexes (24), which limited the number of libraries that can be pooled in a single sequencing experiment. However, pooling libraries representing 96 different genotypes is now possible with the release of a new library preparation kit and should result in an average depth/editing site of 1250.

The sensitivity of RNA-seq allowed us to detect numerous editing sites not reported before (Dataset S3). The limited number of new plastid sites uncovered in this study is not surprising because of the generally less frequent occurrence of editing in plastids vs. mitochondria and because the primers we used did not encompass the entire transcript open reading frames for many of the plastid genes (Table S2). Nevertheless, we found three new plastid sites in the *ndhB* transcript that were not detected in a screen using high resolution melting (HRM) analysis of amplicons [2], demonstrating the superiority of Illumina sequencing of RT-PCR products over HRM analysis. The new picture that arises from our analysis is the existence of numerous mitochondrial sites which are edited to low edited extent, an average of 0.07 in the wild-type accessions assessed. The majority of these new editing sites are silent, which raises the question of their biological significance. It is likely that these sites are secondary targets of the editing machinery which happen to be accidentally edited because of similarity in their putative *cis* element to other *cis* elements found in primary targets of editing (Figure 2). Twelve of the new mitochondrial Arabidopsis sites are found in other species and are therefore edited at sufficient level to have been detected by conventional means. Eleven of these 12 sites are silent (Dataset S3); therefore it is possible that the editing extent at these sites has shifted in certain species due to a lack of selective pressure. We are confident that these new mitochondrial sites are genuine because their occurrence satisfies significance criteria for editing calls based on the mismatch rate in our experiments (Figure S2). The effect of the *rip1* mutation on the editing extent of the new mitochondrial editing sites is the strongest evidence to support their existence. A majority of the new sites, like the previously reported sites, are *rip1*-dependent; moreover, the distribution of *rip1*-dependent sites in the different protein complexes is very similar in both populations (Table 3). If these new sites were false positives, we would not expect them to be subjected to RIP1 control as are most of the reported mitochondrial editing sites.

Numerous mutants in PPR-PLS-motif-containing genes involved in plant organelle editing have been reported to lack any noticeable growth and development phenotype when compared to the wild-type, e.g. [14], [27], despite the absence of detectable editing at one or more C targets. However, because many of these studies relied on bulk sequencing assays, it is possible that the actual extent of editing at some sites could be as high as 10% and go undetected. A residual amount of editing may also affect the phenotype of mutants in the RIP family, explaining why some are viable despite impaired editing of many organelle transcripts [12], [13]. As an example, we found that *rip3-1* and *rip3-2*, two null mutants in the *RIP3* gene, are severely reduced in editing extent of *rps4*-299 (Dataset S1). Editing at this particular site might be important for RPS4 function because it restores a leucine that is found across plant lineages (Figure S5). Nevertheless, the two mutants show only a slight delay in development when compared to wild-type (Figure 1). The residual editing extent of *rp4*-299 detected by Illumina sequencing in *rip3-1* and *rip3-2*, 117 edited transcripts among 12290 transcripts and 71 edited transcripts among 3400 transcripts, respectively (Dataset S1), might be sufficient to encode sufficient functional S4 protein to allow some normal ribosomal activity. Alternatively, despite its evolutionary conservation, perhaps the leucine residue is not absolutely necessary for S4 function. None of the sites whose editing extent is severely reduced in *rip3* mutant plants exhibit a complete absence of edited transcripts (Dataset S1).

### New insight into the function of *RIP* gene family members

We are able to use our method to determine more accurately the effect of altered expression of editing factors due to mutation or silencing on editing extent because of the large number of reads that are obtained for sequences surrounding each editing site. The variation of editing extent, ΔEE = (wild-type – mutant T frequency/wild-type T frequency, is better suited as a metric to measure the effect of a mutation than the difference in editing extent between wild-type and the mutant. For example, the editing of *mttB*-666 was shown to be strongly reduced in the *rip1* mutant (ΔEE = 0.87, Dataset S1) even though this site is poorly edited in the corresponding wild-type (T = 0.04, Dataset S1). Had we considered only the difference of editing extent for *mttB*-666 between the *rip1* mutant and its wild-type sibling (0.04), we would have erroneously considered this site not to be under the control of RIP1.

We have detected that multiple members of the RIP family sometimes affect editing at the same C target. Most of the factors were observed to share editing targets, except for RIP1, for which over 250 mitochondrial sites are solely controlled by this factor (Figures 6 and 7). The fact that more than one factor influences the editing of the same nucleotide suggests that they may interact in the same editing complex. This possibility is consistent with the finding of yeast two-hybrid interaction and with the results of pull-down assays [13]. While another group previously reported that there was almost no overlap in the targets affected by MORF1 (RIP8) and MORF3 (RIP3) [13], with our more sensitive assay we are able to demonstrate that about 55% of the sites under the control of RIP8 are also influenced by RIP3, while about 35% of the sites under the control of RIP3 are also influenced by RIP8 (Figure 6).

RIP1 is rather unique among RIP factors by the sheer number of sites under its control (Table 4) and its dual influence on editing sites in both organelles (Tables 4 and 5). RIP2, which is primarily a plastid editing factor, was the only other family member to have a significant impact on editing in the other organelle (Tables 4 and S6). Based on the dual influence of RIP2 on organelle editing, we checked its targeting by expressing a RIP2-GFP protein in transfected *N. benthamiana* protoplasts. We were unable to find RIP2 in the mitochondria (data not shown), suggesting that the effect of RIP2 on mitochondrial editing might be indirect.

Most of the RIP members that were not characterized before this study are minor editing factors (Table 4). Some of these factors might be involved in other aspects of RNA metabolism. Half of the sites reduced in *rip7*-silenced plants are on the *nad3* transcript, suggesting that RIP7 could control some processes other than editing in the maturation of this transcript (Table S10).

All the wild-type accessions for the *rip* mutant belong to the same genetic background, Columbia, except for *rip1*, which is in the Wassilewskija background. An unexpected level of variation in editing extent was found between the Columbia accessions used in this study, the wild-type siblings of the *rip* mutants and the Columbia used for VIGS. Comparisons of editing extent should therefore be performed between mutants and wild-type siblings whenever possible in order to ensure that the background in the mutants is similar to that of the wild-type. Otherwise, a true reduction in editing extent in the mutant can be obscured by a difference between the genetic background of the mutant and wild-type that affects the wild-type editing extent. The wild-type plants to which the mutants are being compared should also be grown at the same time, in similar conditions. Perhaps some of these considerations explain why we have detected overlapping effects of RIP3 and RIP8 that were not seen by another group [13].

In this study we have analyzed with unprecedented resolution the involvement of a whole family of plant factors in RNA organelle editing. Our data demonstrate a wide range of effect in organelle editing for the RIP proteins, from a major effect for RIP1, a moderate one for RIP2, RIP3, RIP8, and RIP9, to a minor one or none for RIP4, RIP5, RIP6, RIP7 and RIP10. RIP1 can be designated a major component of the editing machinery, as it controls the editing extent of over 50% of the mitochondrial-targeted Cs; it is also the only RIP factor to be dually targeted to both organelles. The important overlapping effects of RIP factors on Cs targeted for editing in mitochondria as well as in plastids support a possible interaction of these proteins in the same editosome complexes. Finally, conservation through evolution of factors such as RIP7 that have only minor effects on editing, combined with a bias in the distribution of the editing targets they control on the organelle transcripts, suggest that these minor factors might be involved in RNA metabolic processes other than RNA editing.

## Materials and Methods

### Plant material

The T-DNA insertion lines were obtained from the ABRC stock center. All plants analyzed for editing extent were grown under long-day conditions of 14 h of light/10 h of dark, under full-spectrum fluorescent lights in a growth room at 26°C. The Col line expressing GFP was kindly donated by Dominique Robertson (North Carolina State University).

With the exception of *rip6-1*, all the mutant populations were segregating so that a wild-type sibling of each mutant was available.

### VIGS

VIGS of *RIP* genes using a GFP co-silencing marker as in [12] were performed for each RIP gene with primers predicted from the Complete Arabidopsis Transcriptome Microarray (CATMA) database [28].

### Library preparation and sequencing

RNA extraction and RT-PCR methods were as described in [3]. Primers to amplify the mitochondrial and plastid transcripts are described in Table S1. After RT-PCR amplification, the 60 amplicons were quantified on a gel and equimolar amount of RT-PCR products (100 fmoles) were mixed for each sample. The mix of organellar cDNA products was then sheared by sonication using a Covaris M220 Focused-ultrasonicator according to the manufacturer recommendations to generate 300 bp fragments. The sheared cDNA (1–2 µg) entered the workflow of the low-throughput protocol for TruSeq RNA Sample Preparation Guide at the step of performing end repair. The following steps to prepare the Illumina libraries were done according to the low-throughput protocol. Three sequencing experiments in which samples s, a, and L were pooled (Table 2) were performed for this study. Prior to sequencing, Illumina libraries were quantified using a nanodrop spectrophotometer (samples s and a) or a Qubit fluorometer (samples L) and mixed in equimolar amounts. The use of different indexes for each library allowed the multiplexing of up to 24 libraries (samples s and a); the correspondence between indexes and samples is given in Table 2. Sequencing was performed with an Illumina/Solexa Genome Analyzer HiSeq 2000 at the Cornell University Life Sciences Core Laboratories Center (one lane/sequencing experiment). To estimate the sequencing mismatch rate, samples L were all spiked with a plasmid template DNA; samples L7, L12, L13 and L14 were spiked with 4 RT-PCR products while samples L15, L16, L18 and L19 were spiked with 4 PCR products. The PCR and RT-PCR products were amplified from the same Arabidopsis nuclear gene specific tags corresponding to *RIP2*, *RIP5*, *RIP8*, and *RIP9* using CATMA primers [28] and a Columbia DNA or RNA template, respectively.

### Read analysis and identification of editing sites

The numbers of Illumina reads obtained for different samples are shown in Table S12. The post-filter (PF) Illumina reads (i.e., the reads passing Illumina's internal quality filter) were pre-processed by trimming 3 first low-quality bases from the 5′ end and clipping the 3′ fragment starting from the first base with Illumina quality below Q20. The read was kept if after these operations its length was at least 60. The pre-processing procedure eliminated about 1–3% of all PF reads, depending on the library, and about 5% of the surviving reads were shorter than 95. The pre-processed reads were aligned to the genomic template using the BWA program [29] with up to 14 mismatches allowed per read. Such a high rate of allowed mismatches was necessary to obtain correct editing ratios for genes with high density of editing sites (*ccmB*, *ccmC*, *mttb*). Keeping only reads with one top alignment, no secondary alignments, and no indels resulted in *ca* 80% of the original PF reads suitable for the analysis. The C->T editing sites were determined for each sample using the likelihood ratio test with error rates computed empirically from alignment data, as described in detail in Protocol S1, in the section “Identification of CT editing sites”. The union of 1833 sites showing significant editing was then constructed over all samples. From among all the sites of the union, we selected a subset of sites having average read depth of at least 100 across all samples and T fraction (T fraction = T/(C+T)) of at least 5% in at least one sample.

### Identification of organelle editing sites exhibiting a reduced editing extent in *rip* mutant and *rip*-silenced plants

The editing extent (T/C+T) between each *rip* mutant plant and its wild-type sibling at a given editing site was tested by a chisquare test with one degree of freedom. χ^2^ = 
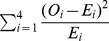
 where *O_i_* = observed frequency, *E_i_* = expected frequency in case of independence. Whenever biological replicates were available for either the mutant or the wild-type, the number of C reads and T reads were pooled together between biological replicates. Because of repetitive testing and its subsequent effect on the familywise error rate, we adjusted the nominal error rate to declare a significant departure from independence by dividing it by the number of tests performed (Bonferroni correction) in order to achieve the desired familywise error rate (P<1e-3). In the case of 619 mitochondrial sites this adjustment results in an error rate of P<1.6e-6; while for the 37 plastid sites the nominal error rate was P<2.7e-5. For *rip*-silenced analysis, the comparison was made between the *rip*-silenced plant and the uninoculated control. Significantly reduced sites in the *rip*-silenced plant were further checked against the GFP-silenced control, because virus inoculation can induce an aspecific effect on editing extent. Only sites showing reduced editing extent in silenced plants against both controls were retained. In addition to satisfying the chi-square test requirement, a site was declared *rip*-dependent if its reduction in editing extent compared to the wild-type (control plants) for the mutant (silenced) was ≥0.1.

### Analysis of RNA editing by PPE

New plastid editing sites were assayed as in [12] with the following primers:

*ndhB*-708: 5′AAGCTTGAACCCAATTCCTACAG

*ndhB*-726: 5′GAGAAGGGGCTAGGGAAAGC

## Supporting Information

Dataset S1 Number of reads at each editing site for each library.
(XLSX)

Dataset S2 Average and standard deviation of editing extent for previously reported sites.
(XLSX)

Dataset S3 Average and standard deviation of editing extent for newly identified sites.
(XLSX)

Figure S1 Typical patterns of read depth along organellar transcripts. The read depth was measured every 10 bp along each cDNA of the *rip1* mutant. (A) *pet L* (B) *rpl16*-trailer (C) *nad1* (D) *atp4*.
(PDF)

Figure S2 Mismatch rates as functions of position on the read derived from alignments of reads to respective templates. The values shown on the vertical axes are *P*_*i* (*o*|*r*) of Eq. (3) (protocol S1), e.g., a curve marked “CA” corresponds to *P*_*i* (*C*|*A*). (A) reads from a plasmid DNA spiked into sample L12 aligned to the plasmid template; (B) reads from PCR products spiked into L19 sample aligned to the PCR DNA templates; (C) reads from RT-PCR products spiked into L12 sample aligned to the RT-PCR templates; (D) reads from L12 sample aligned to the complete organelle genomic templates.
(PDF)

Figure S3 10% editing extent cannot be detected by bulk sequencing. Bulk-sequencing electrophoretograms of RT-PCR products from *rip1* wild-type at sites whose editing extent was determined to be 10% by Illumina sequencing. Above each electrophoretogram is given the editing site; below each electrophoretogram is the Illumina-sequencing-derived editing extent. Notice that no T peak (red) is detectable at the editing site.
(PDF)

Figure S4 Differences in editing extent for some plastid sites in *rip1* and *rip1* wild-type sibling between this study and a previous report are caused by the use of different RNA samples. Inconsistent points between the two studies (green squares) were re-assessed by PPE (red diamonds) on the same RNA samples used for Illumina sequencing. The correlation between PPE and Illumina sequencing was significantly improved (red vs. green) by using the same RNA sample, demonstrating that the discrepancy between the two studies was due to the RNA samples, not the method to measure editing extent.
(PDF)

Figure S5 Alignment of *rps4* sequences in the area upstream to the C at position 299. The C at position 299 is indicated by an arrow and edited to a T in *Arabidopsis thaliana*, *Brassica napus*, and *Citrullus lanatus*. In the other species shown, C is substituted by a genomically-encoded T. Below the DNA sequences is given the amino acid sequence.
(PDF)

Protocol S1 Identification of editing sites and estimation of mismatch rates.
(DOCX)

Table S1 Primers used for organelle RT-PCR.
(XLSX)

Table S2 Comparison of the variation in editing extent for plastid sites significantly controlled by RIP1 (ΔEE>0.1 or ΔEE<−0.1) between this study and previous results.
(XLS)

Table S3 The effects of RIP1 and RIP3 on mitochondrial editing are not independent.
(XLS)

Table S4 The effects of RIP1 and RIP8, and RIP3 and RIP8 on mitochondrial editing are not independent.
(XLSX)

Table S5 Plastid editing sites with editing extent significantly reduced in rip2-silenced plants.
(XLS)

Table S6 Mitochondrial editing sites with editing extent significantly reduced in rip2-silenced plants.
(XLS)

Table S7 Plastid editing sites with editing extent significantly reduced in rip9-silenced plants.
(XLS)

Table S8 The effects of RIP2 and RIP9 on plastid editing are independent.
(XLS)

Table S9 Mitochondrial editing sites with editing extent significantly reduced in both *rip5*-silenced and *rip5-1* mutant plants.
(XLS)

Table S10 Mitochondrial editing sites with editing extent significantly reduced in *rip7*-silenced plants.
(XLS)

Table S11 Minor differences between this study and a previous report.
(XLSX)

Table S12 Quality control of the reads from the different libraries.
(XLS)
