# Supplementary figures and images for: Comprehensive High-Resolution Analysis of the Role of an Arabidopsis Gene Family in RNA Editing
Source: PLoS Genet. 2013 Jun 20;9(6):e1003584. doi: 10.1371/journal.pgen.1003584 (PMC3688494; doi:10.1371/journal.pgen.1003584)

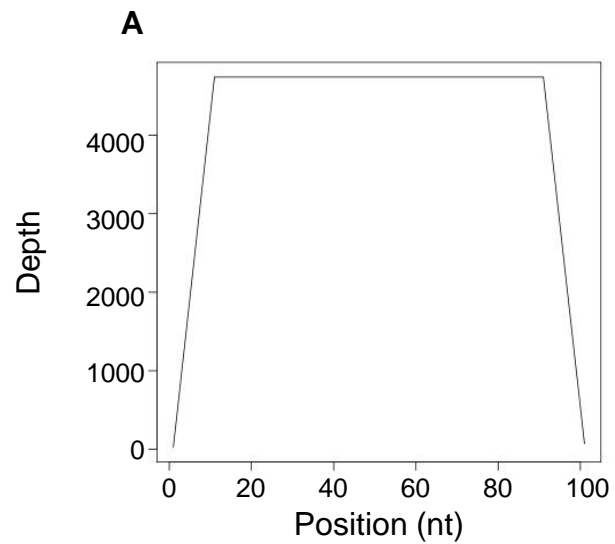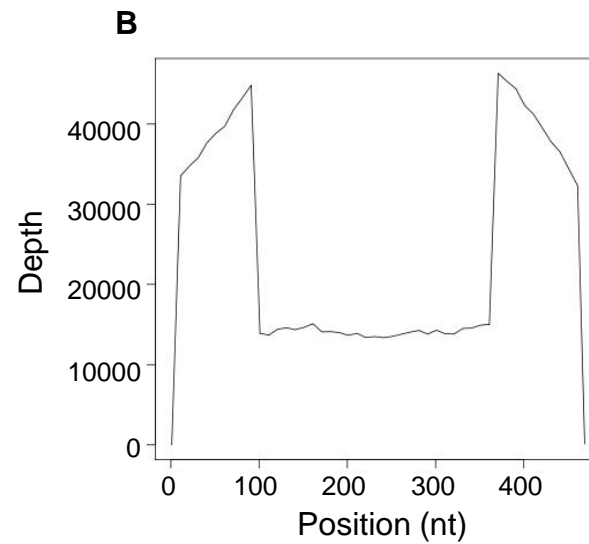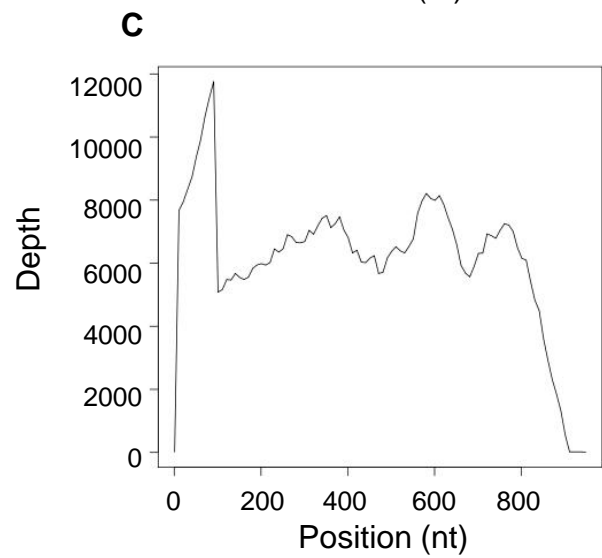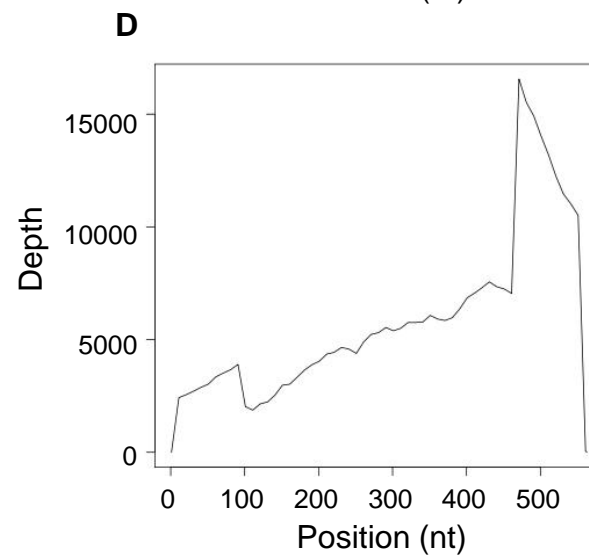

Supplement: Figure S1 — Typical patterns of read depth along organellar transcripts. The read depth was measured every 10 bp along each cDNA of the rip1 mutant. (A) pet L (B) rpl16-trailer (C) nad1 (D) atp4. (PDF) [file pgen.1003584.s004.pdf]

**A**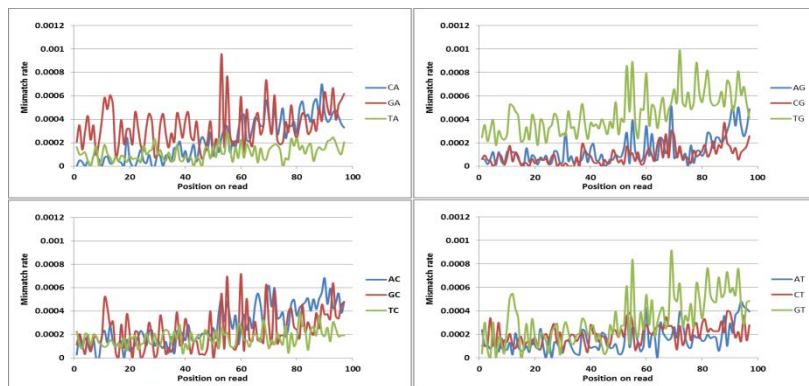**B**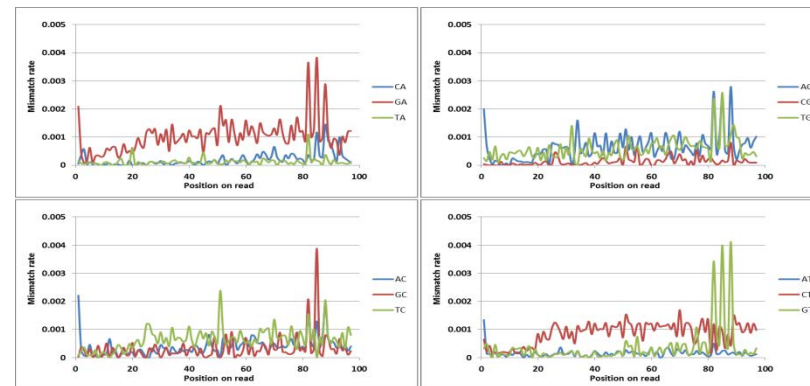**C**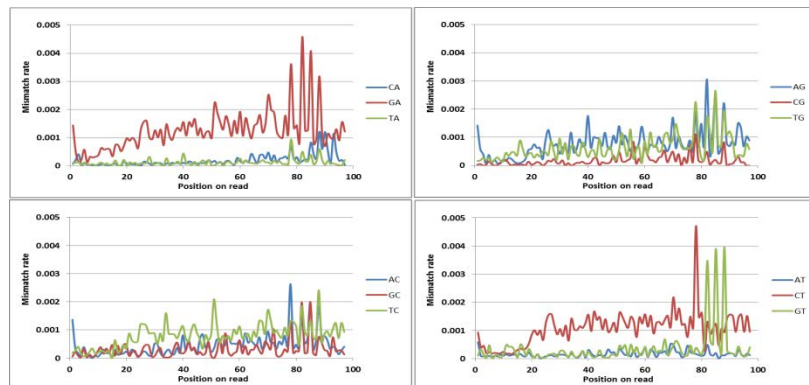**D**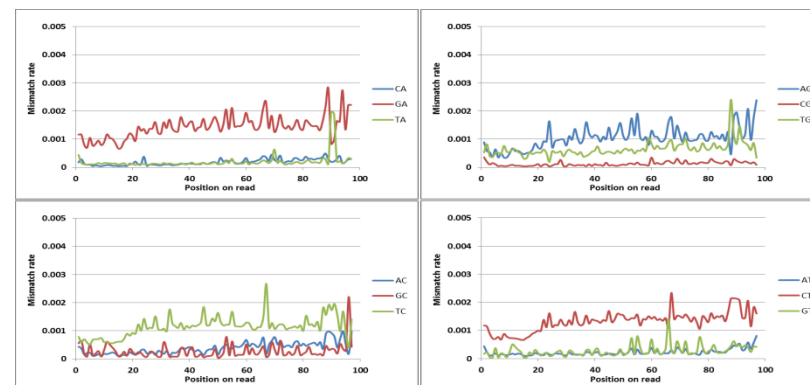

Supplement: Figure S2 — Mismatch rates as functions of position on the read derived from alignments of reads to respective templates. The values shown on the vertical axes are P_i (o|r) of Eq. (3) (protocol S1), e.g., a curve marked “CA” corresponds to P_i (C|A). (A) reads from a plasmid DNA spiked into sample L12 aligned to the plasmid template; (B) reads from PCR products spiked into L19 sample aligned to the PCR DNA templates; (C) reads from RT-PCR products spiked into L12 sample aligned to the RT-PCR templates; (D) reads from L12 sample aligned to the complete organelle genomic templates. (PDF) [file pgen.1003584.s005.pdf]

*ccmC*-655

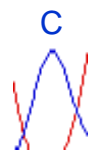

0.10

*cox2*-135

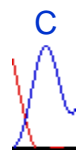

0.10

*nad2*-642

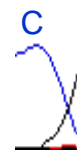

0.10

*nad2*-888

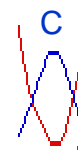

0.10

*rpl2*-292

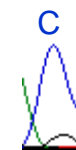

0.10

Supplement: Figure S3 — 10% editing extent cannot be detected by bulk sequencing. Bulk-sequencing electrophoretograms of RT-PCR products from rip1 wild-type at sites whose editing extent was determined to be 10% by Illumina sequencing. Above each electrophoretogram is given the editing site; below each electrophoretogram is the Illumina-sequencing-derived editing extent. Notice that no T peak (red) is detectable at the editing site. (PDF) [file pgen.1003584.s006.pdf]

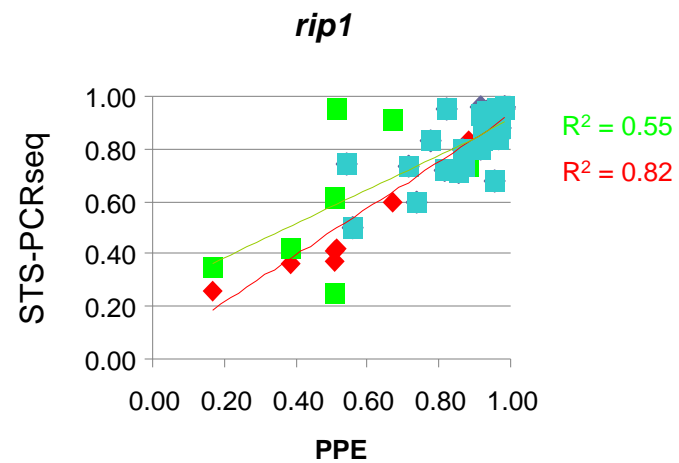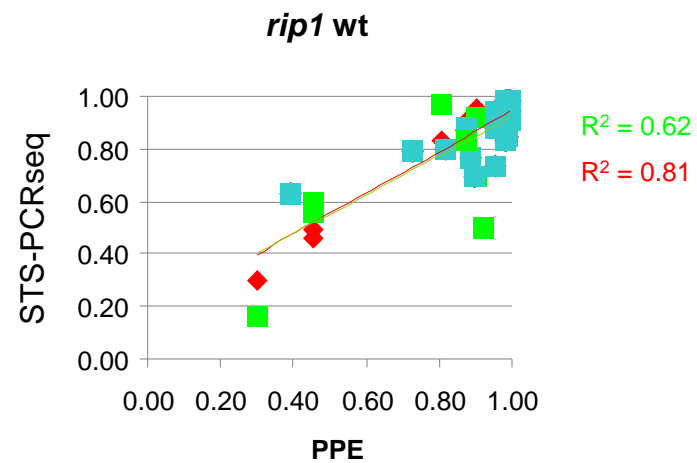

Supplement: Figure S4 — Differences in editing extent for some plastid sites in rip1 and rip1 wild-type sibling between this study and a previous report are caused by the use of different RNA samples. Inconsistent points between the two studies (green squares) were re-assessed by PPE (red diamonds) on the same RNA samples used for Illumina sequencing. The correlation between PPE and Illumina sequencing was significantly improved (red vs. green) by using the same RNA sample, demonstrating that the discrepancy between the two studies was due to the RNA samples, not the method to measure editing extent. (PDF) [file pgen.1003584.s007.pdf]
