# Supplementary material for: Comprehensive High-Resolution Analysis of the Role of an Arabidopsis Gene Family in RNA Editing
Source: PLoS Genet. 2013 Jun 20;9(6):e1003584. doi: 10.1371/journal.pgen.1003584 (PMC3688494; doi:10.1371/journal.pgen.1003584)
Supplement: Figure S5 — Alignment of rps4 sequences in the area upstream to the C at position 299. The C at position 299 is indicated by an arrow and edited to a T in Arabidopsis thaliana, Brassica napus, and Citrullus lanatus. In the other species shown, C is substituted by a genomically-encoded T. Below the DNA sequences is given the amino acid sequence. (PDF) [file pgen.1003584.s008.pdf]

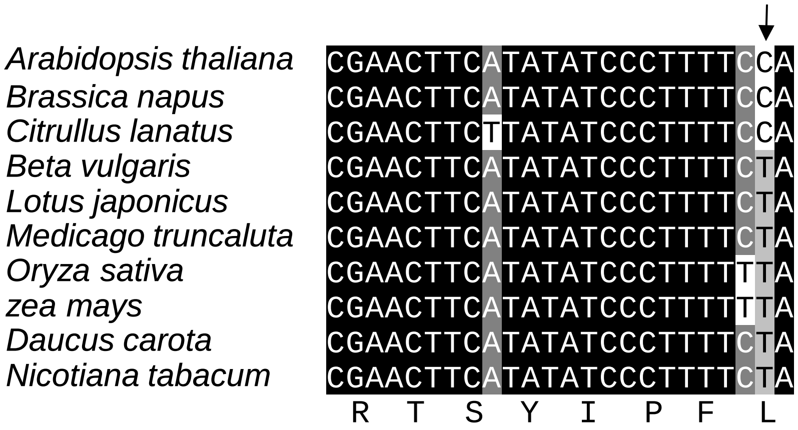

Arabidopsis thaliana CGAACTTCATATATCCCTTTTCCA
Brassica napus CGAACTTCATATATCCCTTTTCCA
Citrullus lanatus CGAACTTCTTATATCCCTTTTCCA
Beta vulgaris CGAACTTCATATATCCCTTTTCTA
Lotus japonicus CGAACTTCATATATCCCTTTTCTA
Medicago truncaluta CGAACTTCATATATCCCTTTTCTA
Oryza sativa CGAACTTCATATATCCCTTTTTTA
zea mays CGAACTTCATATATCCCTTTTTTA
Daucus carota CGAACTTCATATATCCCTTTTCTA
Nicotiana tabacum CGAACTTCATATATCCCTTTTCTA
R T S Y I P F L
