# Supplementary material for: Comprehensive High-Resolution Analysis of the Role of an Arabidopsis Gene Family in RNA Editing
Source: PLoS Genet. 2013 Jun 20;9(6):e1003584. doi: 10.1371/journal.pgen.1003584 (PMC3688494; doi:10.1371/journal.pgen.1003584)
Supplement: Protocol S1 — Identification of editing sites and estimation of mismatch rates. (DOCX) [file pgen.1003584.s009.docx]

# Identification of CT editing sites

For each genomic position with reference base C, we used the likelihood ratio test to assess how well the alignment data was explained by an assumption that the reads originated from a template mixture containing C and T alleles at certain proportion. We defined the likelihood function

|  | $L\left( D \vert CT;\omega\right)= \prod_{o} P_{i}(o\vert CT;\omega)$ |  |
| --- | --- | --- |

where $D$ denotes the pileup of all bases $o$ aligned at this genomic position, $i$ denotes the position of base $o$ on the read, and $\omega$ is the CT edit fraction. The probability of observing base $o$ given edit fraction $\omega$ is given by

|  | $P_{i}\left( o \vert CT;\omega\right)=\left( 1-\omega\right)P_{i}\left( o \vert C \right)+ \omega P_{i}(o\vert T)$ |  |
| --- | --- | --- |

where $P_{i}(o|r)$ is the probability of observing base $o$ on read position $i$ given the template the read originated from had base $r$ at the genomic position which $i$ maps to. For the latter probability, the following model was assumed

|  | $P_{i}\left( o \vert r \right)=\left\{ \begin{aligned} 1-e_{i} \mathrm{if} o=r \\ e_{i}\varepsilon_{or}^{i} \mathrm{if} o\neq r \end{aligned} \right.$ |  |
| --- | --- | --- |

where the overall mismatch rate $e_{i}$ and the confusion matrix $\varepsilon_{or}^{i}$ were estimated from the data as functions of the position $i$ on the read as described below. When the likelihood $L\left( D | CT;\omega\right)$ is maximized over $\omega$ and compared to the likelihood of data under the condition$\omega$=0 (no edit), then the quantity

|  | $d=-2\ln\left( \frac{L(D\vert CT;\omega=0)}{\max_{\omega} L(D\vert CT;\omega)} \right)$ |  |
| --- | --- | --- |

has the $\chi^{2}$distribution with one degree of freedom. Similarly to $L\left( D | CT;\omega\right)$ one can also define likelihoods of data under the assumption that the reads originated from a mixture of C and one of the other (non-T) bases: $L\left( D | CA;\alpha\right)$ and$L\left( D | CG;\beta\right)$, with $\alpha$ and $\beta$ denoting the A and G fraction in the template mixture, respectively. The CT edit was called at a genomic position with reference base C if the major allele was T or if the major allele was C and the following two conditions were satisfied:

1. $\max_{\omega} L(D|CT;\omega)>\max_{\alpha} L\left( D | CB;\alpha\right)$, where $B$ is the second (besides $T$) minor allele observed
2. p-value of $d$ derived assuming $\chi^{2}(1)$ distribution was below the threshold of${10}^{-6}$ .

The p-value threshold was chosen as ${10}^{-6}$ to ensure that the probability of any CT edit being called accidentally stays below 1% (applying Bonferroni correction for repetitive testing with 8320 C sites gives 0.01/8320 = $1.2\times{10}^{-6}$). All calculations were performed using the mpileup function of the samtools package [34] coupled with custom perl scripts. Likelihood maximizations were performed using a numerical procedure.

# Estimating mismatch rates

The mismatch rates used in Eq. (3) have been obtained for each sample individually from alignment of this sample’s reads to the respective template. Pre-processing of reads and alignment were performed as described in Section “Read analysis and identification of editing sites”. For each position on the reference, pileup of aligned bases was obtained using samtools [34]. Each base in the pileup was compared to the reference base and counts of matches and mismatches were collected over genomic positions. These counts were binned as functions of position on the read. For each fixed position $i$ on read, the overall mismatch rate was calculated as

|  | $e_{i}= \frac{\sum_{o,r\neq o} c_{i}(o\vert r)}{\sum_{o,r} c_{i}(o\vert r)}$ |  |
| --- | --- | --- |

and the confusion matrix was given by

|  | $\varepsilon_{or}^{i}=\frac{c_{i}(o\vert r)}{\sum_{o\neq r} c_{i}(o\vert r)}$ |  |
| --- | --- | --- |

where $c_{i}(o|r)$ denotes the count of base $o$ observed at read position $i$ given the reference base was $r$. Note that $\sum_{o\neq r} c_{i}(o|r)=1$, so that also $\sum_{o} P_{i}\left( o | r \right)=1$, as expected.

While collecting mismatch counts for error estimates one needs to exclude genomic positions with known polymorphisms, such as highly edited C sites or obvious SNPs. A site was excluded if the number of reads mapping to it was at least 100 and one of the following conditions was satisfied:

1. Major allele was different from reference allele
2. Reference and major alleles were C, the first minor allele was T, and depth of the T allele was more than 10% of the combined depth of C and T alleles

Variation at all other genomic sites was considered erroneous and contributed to mismatch rate estimates. Once these estimates were used to call CT edits as described above, some of these sites turned out to be statistically significant edits. Mismatch rates obtained in this way reflect errors arising from the experimental procedure to the extent allowed by the adopted strategy of read pre-processing and alignment.
